# 7-*O*-methylpunctatin is a potential inhibitor of human arachidonate 5-lipoxygenase: molecular and structural insights into anti-atherosclerosis therapeutics

**DOI:** 10.1007/s11030-025-11420-2

**Published:** 2026-01-06

**Authors:** Ghazi Elamin, Ali H. Eid

**Affiliations:** 1 Department of Pharmaceutical Chemistry, College of Pharmacy, Karary University, 11111 Khartoum, Sudan; 2 Department of Basic Medical Sciences, College of Medicine, QU Health, Qatar University, Doha, Qatar

**Keywords:** Atherosclerosis, Homoisoflavonoids, 7-*O*-methylpunctatin, *In silico*, ALOX5

## Abstract

We have recently demonstrated that 7-*O*-methylpunctatin (MP), a novel homoisoflavonoid, suppresses inflammation-induced arterial pathogenesis. However, the precise biochemical mechanisms underlying its atheroprotective effects remain elusive. In this study, we employed various in silico studies to elucidate MP’s plausible potential and the specific molecular pathways through which it exerts its influence on atherosclerosis. Our analysis of MP’s pharmacokinetic, physicochemical, and toxicological properties revealed a profile characterized by favorable absorption, efficient metabolism and excretion, and minimal toxicity. Through target identification and protein-protein interaction analyses, we identified ALOX5 as a pivotal hub gene—an enzyme critically involved in the pathogenesis of atherosclerosis. Furthermore, we identified ten transcription factors and four kinases as potential targets. Molecular mechanics/generalized-born surface area calculations, complemented by time-scale molecular dynamics simulations, revealed that MP binds to ALOX5 with high affinity, modulating its structural stability, rigidity, compactness, overall folding pattern, and residual correlations and motions. These findings corroborate previous in vitro and in vivo investigations that underscore the anti-atherosclerotic effects of ALOX5 inhibition, thereby positioning MP as a promising therapeutic candidate for combating atherosclerosis.

## Introduction

Atherosclerosis is a progressive disease characterized by the build-up of atheromatous plaques within the arterial walls [1]. This, combined with the accompanying inflammation, often leads to vascular narrowing and reduced blood flow [2]. Several factors such as hyperlipidemia, hypertension, smoking, and chronic inflammation instigate this pathological process [3–6]. Over time, the infiltration of low-density lipoprotein (LDL) into the intima and its subsequent oxidation elicit an immune response, promoting the recruitment of monocytes and their differentiation into foam cells [7]. The resulting plaques and the ensuing arterial stenosis impair organ perfusion and predispose to acute events such as myocardial infarction, stroke, and peripheral arterial disease [8]. These conditions increase the morbidity and mortality burden of cardiovascular disease (CVD), of which atherosclerosis remains a major contributor [9, 10]. Thus, atherosclerosis remains both a central focus of cardiovascular research and a critical target for novel therapeutic interventions.

**Fig. 1:**
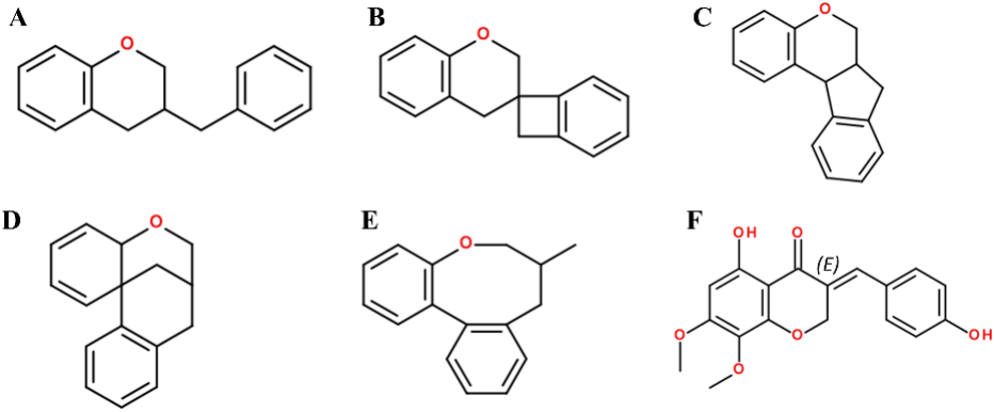
Carbon skeletons of various subtypes of homoisoflavonoids. **A** Sappanin, **B** Scillascillin, **C** Brazillin, **D** Caesalpin, **E** Protosappanin and **F** 7-*O*-methyldropunctatin

In response to the limitations of conventional pharmacotherapies, increasing attention has turned toward plant-derived or inspired molecules that could play an important role in the amelioration of several diseases including CVD [11–20]. To that end, several classes have been identified, prime of which are flavonoids which indeed represent a particularly versatile family of phenolic metabolites possessing potent anti-oxidant and anti-inflammatory characteristics [12–14]. Within this class, homoisoflavonoids, characterized by a distinctive 3-benzylidene-4-chromanone backbone, have emerged as promising molecular scaffolds due to their structural diversity and pharmacological range [21]. These compounds encompass several subclasses, including Sappanin, Scillascillin, Brazilin, Caesalpin, and Protosappanin, each possessing specific physiochemical and biological properties (Fig. 1). Sappanin derivatives exhibit strong hydrogen bonding and π–π stacking interactions that may enhance target affinity and stability. In contrast, Scillascillin-type homoisoflavonoids bear C-glycosyl substituents, which are implicated in enhanced chemical stability and aqueous solubility. Brazilin analogues exhibit a greater degree of conformational flexibility alongside intrinsic redox activity, while the Caesalpin subgroup is defined by a rigid, planar scaffold conducive to improved membrane permeability. Protosappanin derivatives, possessing oxidized core structures are hypothesized to favor polar intermolecular interactions that may facilitate blood-brain barrier traversal. These molecular distinctions underpin the capacity of homoisoflavonoids to exert antioxidant, anti-inflammatory, and cardio-vasculoprotective effects [21–26], positioning them as valuable candidates for mitigating vascular injury and atherogenesis.

Accumulating evidence provides the *raison d’etre* of utilizing these bioactives in the fight against atherosclerosis [21–26]. For instance, they can scavenge reactive oxygen species and modulating pro-inflammatory signaling pathways [21–26]. As such, homoisoflavonoids may mitigate the oxidative damage and vascular inflammation that is not permissive but also instigative to atherogenesis [21–26]. Furthermore, their ability to favourably modulate lipid metabolism and inhibit smooth muscle cell proliferation suggests multifaceted protection across the vascular wall. These properties converge to attenuate both the initiation and propagation of atherosclerotic lesions, offering a pharmacological coherent basis for translational exploration.

Building on this rationale, recent findings from our group identified 7-O-methyldihydropunctatin (MP), a novel homoisoflavonoid that distinctively carries a unique 7-methoxy group which increases molecular lipophilicity, enhances systemic bioavailability, and augments binding affinity to biological targets. We have previously shown that MP potently suppresses vascular smooth muscle cell (VSMC) phenotypic switch from a contractile to a synthetic state [23]. Given that phenotypic modulation of VSMCs is a central determinant of plaque stability and progression [8, 15, 18, 23, 27–29], elucidating the molecular targets and signalling pathways engaged by MP is essential. This study, therefore, was undertaken to identify the potential targets with which MP may interact, thereby modulating the signaling cascades implicated in the pathogenesis of atherosclerosis.

## Materials and methods

## Computational methodology

### ADME and toxicity prediction of MP

The pharmacokinetics of the MP [23, 30], have been investigated using SwissADME [31], a web-based tool that assesses the functional properties of hit substances within the body. Toxicological evaluations of substances are also a crucial aspect of developing new drugs. This enables the identification of a compound’s toxic characteristics via the calculated lethal dose ($\:{\mathrm{L}\mathrm{D}}_{50}$) in mg/kg body weight. The ProTox-II web server was used to calculate the toxicity of chemicals by combining fragment propensity, machine learning, and molecular similarity [32]. The substances are categorized from class one to six depending on their safety, referring to the worldwide harmonized system for categorizing and labelling chemicals [33, 34].

### Target identification

Drug design and development rely on discovering possible targets for a bioactive substance that has already been identified [35]. Conventional methods for accurately identifying potential targets encompass protein affinity separation followed by mass spectrometric study and methods reliant on mRNA expression [36]. However, experimental techniques are costly and time-consuming, rendering in silico target identification a viable alternative for target identification due to these limitations [35]. Ligand-based approaches were conducted through 2D, 3D, or machine learning techniques [37]. Target identification was conducted using SwissTargetPrediction, a web-based platform that predicts potential biological targets of small molecular by virtue of chemical similarity, thereby facilitating the recognition of plausible drug-target interactions for novel compounds [38]. Targets cross-validation was performed using SuperPred 3.0 [39], a platform that integrates machine learning and chemical similarity algorithms for target prediction and drug repurposing, thereby aiding in the inference of potential targets and therapeutics indications. In parallel, the PASS Online web resource was employed to predict biological activity spectra based on compound structure, thus enabling preliminary assessment of pharmacological potential and putative toxicities [40]. Furthermore, the InteractiVenn online application [41], was utilized to construct a Venn Diagram integrating an accessible atherosclerosis-related dataset obtained from the DisGeNET database—a comprehensive repository that interlinks genes, variants, and diseases, thereby supporting drug discovery by connecting genetic insights with underlying disease mechanisms [42, 43].

### Protein–protein interaction of candidate targets

The identified potential targets were submitted to the STRING database v12.0 (https://string-db.org/) for the analysis of protein–protein interactions (PPI). The STRING platform serves as rigorously curated repository encompassing up-to-date information on both physical and functional protein associations [44]. Advanced parameters were configured as follows: organism—*Homo sapiens*; network type—full STRING network; required confidence score—medium (0.7); and false discovery rate (FDR) stringency—medium (5%). The resulting PPI network was subsequently analyzed using the CytoNCA plugin in Cytoscape 3.9.1, taking into account centrality metrics including degree, betweenness, closeness, and eigenvector values [45, 46].

### Analysis of upstream regulatory networks

The eXpression2Kinases (X2K) a web-based platform was employed to generate a PPI network and identify key upstream transcription factors regulating the differentially expressed genes (DEGs) [47]. The initial phase in the X2K workflow involved Transcription Factor Enrichment Analysis (TFEA), designed to identify transcription factors likely controlling the DEGs through ChIP-seq-based gene set enrichment analysis (ChEA). Subsequently, the Genes2Networks (G2N) algorithm was used to expand the set of enriched transcription factors through PPI expansion, identifying proteins that physically interact with these transcriptional regulators. The final stage comprised Kinase Enrichment Analysis (KEA), which used kinase-substrate interaction databases to perform enrichment analysis on the protein list derived from the PPI network, thereby elucidating kinase-mediated regulatory axes.

### System preparation and molecular docking

The X-ray crystal structure of the selected enzyme, Arachidonate 5-lipoxygenase (ALOX5), was retrieved from the RCSB Protein Data Bank [48], using the corresponding PDB code 3V98 [49]. The structure had been resolved by X-ray diffraction at a resolution of 2.7 Å resolutions. The crystal structure of apo-ALOX5 reveals an N-terminal β-barrel domain along with a C-terminal catalytic domain that harbors a non-heme iron coordinated by His367, His372, and His550. Key residues, including Leu420, Phe421, Ile673, Asn554, and Trp599, define the hydrophobic substrate channel and stabilize arachidonic acid, providing important structural insights into leukotriene biosynthesis and facilitating rational inhibitor design. Structure preparation for molecular dynamics (MD) simulation was subsequently carried out using the UCSF Chimera software suite [50], and Molegro Molecular Viewer (MMV) [51]. Our computational analysis focused on the very important chain A of ALOX5, which constitutes the principal catalytic domain. Missing residues in chain A were reconstructed using Modeller [52], a structural refinement tool.

The Molecular structure of MP that we previously reported [23] was generated using MarvinSketch version 19.2 [53]. Zileuton, a well-established inhibitor of ALOX5, served as a reference ligand, with its structural data (ID: 60490) retrieved from PubChem [54–57]. This was followed by a geometric optimization of the structures using Avogadro [58]. The optimized conformers of MP and Zileuton were subsequently prepared for docking into the active site of ALOX5, which was delineated according to co-crystallized ligand data from prior structural studies [59].

Before docking, the ALOX5 protein structure was examined using UCSF Chimera, wherein all cofactors, non-standard residues (Cl-, Na+, etc.), and water molecules were removed to minimize structural distortion during docking and to ensure a stable binding pose [50, 58]. AutoDock Tools GUI was employed for docking computations and conformational sampling was conducted using the Lamarckian Genetic algorithm, noted for its efficiency and reliability in exploring binding landscapes [60, 61]. The grid box was defined with dimensions x = 15.59, y = 11.94, z = 5.98, and centered at x= − 37.4197, y = 91.185, z = 12.2135.

MD simulations were subsequently performed on the complex exhibiting the most favourable (i.e. highest negative) binding energy (kcal.mol-1). Three systems were established for comparative simulation: (i) the unbound (Apo) ALOX5 structure, (ii) the ALOX5-MP complex, and (iii) the ALOX5-Zileuton complex. System refinement was achieved using Maestro Schrödinger, which optimized protonation states, adjusted critical hydrogen orientations, and capped neutral residues to preserve protein stability throughout the simulations [62–64]. The ligand-free ALOX5 system was further examined following established simulation protocols extensively utilized in our prior research [65–67].

## MD simulations

### Simulations protocol

The AMBER18 software and its Particle Mesh Ewald Molecular Dynamics (PMEMD) module were used to conduct MD simulations on unbound (apo) ALOX5 and its complexes with MP or Zileuton [68, 69]. The correct charge state was applied to protein systems that were modelled using the standard Amber (FF14SB) force field that is part of the Amber package [70, 71]. Modifying, renaming, and protonating (histidine) ALOX5 was carried out using an in-house pdb4amber script. Subsequently, the LEAP module was employed to generate ALOX5 parameters and topology files, as well as to neutralize the overall charge.

MD simulations provide atomistic insight into the temporal evolution of biomolecular systems by solving Newton’s equations of motion for all atoms in the system [72, 73]. In this approach, interatomic forces are computed based on a defined potential energy function derived from the force field, allowing continuous monitoring of conformational transitions, binding interactions, and dynamic stability under near-physiological conditions [74–76]. The PMEMD module enhances computational efficiency by using the Particle Mesh Ewald method for long-range electrostatics, thereby enabling accurate treatment of periodic boundary conditions and solvent interactions.

Energy minimization was performed in two stages: an initial restrained minimization with a harmonic constraint of 500 kcal/mol, followed by 2500 steps of partial minimization and 5000 steps of full minimization to relieve steric clashes. The systems were then subjected to gradual thermal equilibration from 0–300 K under constant volume conditions. The atmospheric pressure was maintained at 1 bar using a Berendsen barostat, and the unconstrained equilibration was carried out at 300 K for 1000 ps to ensure thermodynamic stability [77]. Ultimately, a 500-ns production MD simulation was performed for each system to explore the conformational and dynamic effects of MP and Zileuton binding on the structural integrity of ALOX5 [78].

### Post-MD simulation analysis

The trajectories of ALOX5, both in its unbound ALOX5 (apo) state and when complexed with MP or zileuton, were analyzed using the CAPTRAJ modulate integrated within AMBER18, with trajectory frames recorded at 1-ps intervals [79]. Following completion of MD simulation, a comprehensive assessment of system properties was undertaken, encompassing root mean square deviation (RMSD) for overall structural stability, root mean square fluctuation (RMDF) for residue-level flexibility, radius of gyration (Rg) for molecular compactness, and solvent-accessible surface area (SASA) for conformational exposure. In addition, intermolecular hydrogen bonding (HB) and dynamic cross-correlation matrices (DCCM) were examined to elucidate both structural cohesion and correlated atomic motions within the complexes.

To further characterize conformational dynamics, principal component analysis (PCA) was employed to quantify the dominant modes of atomic displacement and collective motion, thereby revealing high-scale conformational transitions induced by ligand-binding. The Molecular Mechanics/Generalized Born Surface Area (MM/GBSA) approach was then used to evaluate ligand-protein binding free energies, providing thermodynamic insight into the stability and favorability of the ALOX5-MP and ALOX5-Zileuton complexes. Visualization and quantitative analyses of trajectories and energetic profiles were conducted using Microcal Origin [80], Discovery Studio (BIOVIA 2017) [81], and NMWiz integrated into Visual Molecular Dynamics (VMD) [82]. Collectively, these analyses afforded an intricate depiction of the conformational landscape and energetic dynamics of ALOX5, by virtue of which ligand-induced modulation could be discerned with atomic precision.

### Calculations of the free energy of protein-ligand interactions using MM/GBSA

The binding free energies, along with their contributing components, namely electrostatic, van de Waals, polar solvation, and non-polar solvation energies, were computed for the ALOX5-MP and ALOX5-Zileuton complexes using the MM/GBSA methodology [83–86]. This approach provides a semi-empirical estimation of the total free energy of binding by decomposing the interactions into physically meaningful terms, offering valuable insight into the driving forces that stabilize ligand association within the active site.

The MM/GBSA equation can be expressed mathematically as:

i $$ \Delta {\mathrm{G}}_{{{\mathrm{bind}}}} = {\text{ G}}_{{{\mathrm{complex}}}} {-}{\text{ G}}_{{{\mathrm{protein}}}} {-}{\text{ G}}_{{{\mathrm{inhibitor}}}} $$

ii $$ \Delta {\mathrm{G}}_{{{\mathrm{bind}}}} = {\text{ E}}_{{{\mathrm{gas}}}} + {\text{ G}}_{{{\mathrm{sol}}}} {-}{\text{ TS}} $$

The ΔG_bind_ is the sum of the gas and solvent energy minus entropy (TS).

iii $$ {\mathrm{E}}_{{{\mathrm{gas}}}} = {\text{ E}}_{{{\mathrm{int}}}} + {\text{ E}}_{{{\mathrm{vdw}}}} + {\text{ E}}_{{{\mathrm{ele}}}} $$

In this context, E_gas_ represents the total internal energy of the AMBER force fields, which include E_int_ for bond, torsion, and angle energies; E_vdw_ for van der Waals energies related to covalent bonds; and E_elec_ for electrostatic energies related to non-bonded pairs.

The equation used for calculating the solvent energy is as follows:

iv $$ {\mathrm{G}}_{{{\mathrm{sol}}}} = {\mathrm{G}}_{{{\mathrm{GB}}}} + {\text{ G}}_{{{\mathrm{SA}}}} $$

v $$ {\mathrm{G}}_{{{\mathrm{SA}}}} = {\text{ }}\gamma {\mathrm{SASA}} $$

G_GB_ stands for the polar solvation effect and G_SA_ for the non-polar solvation effect, both calculated using SASA. This is attainable by the 1.4 A° water probe combined with ‘b’ surface tension constant values of 0 kcal/mol and ‘c’ values of 0.0072 kcal/mol [87]. Finally, to investigate ALOX5’s stability, a per-residue energy decomposition analysis was carried out to determine the possible energy contributions of certain residues inside the catalytic region. This provides an atomic-level understanding of MP and Zileuton blocking potential and sheds light on the mechanism of ALOX5 targeting, as shown by MP and Zileuton binding.

### Dynamic cross-correlation matrices (DCCM)

The analysis of dynamic cross-correlation of MD simulation trajectories was employed for investigating the fluctuations and movements in the backbone of the α-carbon atoms of ALOX5 [88]. Cross-correlation components for i and j Cα atoms are shown in Eq. 1:

1 $$ {\mathrm{C}}_{{{\mathrm{ij~}}}} = {\mathrm{~}}\frac{{\left\langle {\Delta {\mathrm{ri}}.\Delta {\mathrm{rj}}} \right\rangle }}{{\left( {\left\langle {\Delta {\mathrm{r}}_{{\mathrm{i}}}^{2} } \right\rangle \left\langle {\Delta {\mathrm{r}}_{{\mathrm{j}}}^{2} } \right\rangle } \right)^{{\frac{1}{2}}} }} $$

Where r_i_ = Cα^i^ = standard time throughout the MD trajectory. Significant correlations in motions are denoted by Cij = 1, whereas Cij = − 1 indicates strong anticorrelations in the trajectory. The motion deviation from 1 to − 1 signifies that the movements of I and j are anti-correlated.

### Principal component analysis (PCA)

The ALOX5 residual motions in free state (Apo) and when bound to the MP and Zileuton have been revealed by PCA, an effective method for studying protein structural changes [89]. PCA uses MD trajectories to generate highly correlated and anticorrelated fluctuations through dimensional reduction [90]. The atomic coordinates and eigenvectors were utilized to produce a positional covariance matrix C, subsequently employed to analyze collective motions. The eigenvalues indicate the extent of the motion, whereas the eigenvectors indicate the direction of the motion [91]. The positional covariance matrix C was calculated using the equation provided:

2 $$ {\mathrm{C}}_{{\mathrm{i}}} ~ = ~\left\langle {\left( {{\mathrm{q}}_{{\mathrm{i}}} - \left\langle {{\mathrm{q}}_{{\mathrm{i}}} } \right\rangle } \right)\left( {{\mathrm{q}}_{{{\mathrm{j}}~~}} - \left\langle {{\mathrm{q}}_{{\mathrm{j}}} } \right\rangle } \right)} \right\rangle {\kern 1pt} \,\;~\left( {~{\mathrm{i}},{\mathrm{j}}~ = 1,2,~ \ldots ,~3{\mathrm{N}}} \right) $$

The variables qi and qj represent the Cartesian coordinates of the i-th and j-th Cα atoms, respectively, and N denotes the total number of Cα atoms. The average is calculated after superimposing the MD trajectories with a reference structure using a least-square fit approach to extract the essential motion, so excluding all translational and rotational movements [92]. The eigenvalues and eigenvectors are computed by performing an orthogonal coordinate transformation on the symmetric matrix C, resulting in a diagonal matrix Λ of eigenvalues, as demonstrated below:

3 $$ \wedge = {\mathrm{~T}}^{{\mathrm{T}}} {\mathrm{~C}}_{{{\mathrm{ij~~}}}} {\mathrm{T}} $$

The eigenvalues here indicate the system’s total mean-square variation along each eigenvector, and the eigenvectors point in the directions of motion to (qi).

## Results and discussion

### Physicochemical and toxicity properties and Drug-likeness of the MP

Adverse physiological outcomes represent a central concern in the clinical translation of newly discovered therapeutic agents. Therefore, a comprehensive evaluation of pharmacokinetic, physicochemical and toxicological attributes is indispensable for delineating a compound’s metabolic efficacy and safety profile [93]. The interplay between molecular structure and biological disposition remains a defining determinant of drug performance. Indeed, it has long been established that an increase in molecular weight attenuates a compound’s concentration gradient across the intestinal epithelial interface, thereby diminishing passive absorption efficiency [94].

Such physicochemical constraints may, by virtue of steric and diffusion limitations, hinder transbilayer permeability, consequently impairing bioavailability and therapeutic potency. To mitigate this, Lipinski’s Rule of Five (RO5) serves as a foundational framework for assessing drug-likeness, postulating that orally active candidates should generally exhibit a molecular weight below 500 g/mol, no more than five hydrogen bond donors, ten hydrogen bond acceptors, and a logP not exceeding five [95, 96].

Within this context, MP conformed to the molecular weight criterion stipulated by RO5, thereby aligning with the physicochemical prerequisites of drug-like compounds. As shown in Table 1, MP’s molecular characteristics suggest favorable pharmacokinetic behavior and an acceptable absorption profile, affirming its potential as a viable drug candidate.

**Table 1:** Physiochemical and toxicity properties of the MP

Physiochemical properties	
Formula	C18H16O6
MW (g/mol)	328.32
MLog P _O/W_	0.82
Log S (Ali) (mol/L)	− 4.58
Class	Moderately soluble
TPSA (Å²)	85.22
Molar Refractivity	87.66
H-bond acceptors	6
H-bond donors	2
Rotatable bonds	3
Lipinski violations	Yes; 0 violation

Toxicity	
LD_50_ (mg/kg)	2652
Toxicity class	5

MW = molecular weight (g/mol) (MW ≤ 500); MLOGP = predicted octanol/water partition coefficient (MLOGP < 4.15); TPSA = Topological polar surface area (≤ 140); HBA = number of hydrogen bond acceptors (HBA ≤ 10); HBD = number of hydrogen bond donors (HBD ≤ 5); Rotatable bonds ≤ 8

To further elucidate the influence of molecular weight on bioactivity, the number of rotatable bonds provides a robust indicator of molecular flexibility, as an increased number of such bonds often correlates with higher molecular weight and reduced molecular rigidity [97, 98]. Excessive flexibility may impede optimal receptor binding and membrane permeability. According to Lipinski’s RO5, drug-like molecule should possess fewer than ten rotatable bonds to ensure adequate absorption and bioavailability [97, 99, 100]. Our computational assessment revealed that MP exhibited three rotatable bonds, well within the permissible range, suggesting favourable molecular flexibility conducive to efficient pharmacokinetic performance.

The partition coefficient (LogP), defined as the logarithm of the concentration ratio of a compound between *n*-octanol and water (C_Octanol_/ C_Water_), serves as a critical measure of hydrophobicity and lipophilicity [101]. Elevated MLogP values imply reduced aqueous solubility and, by virtue of this, diminished intestinal absorption potential. Mp displayed a MLogP value of 0.82, denoting balanced hydrophilic-lipophilic characteristics suitable for oral bioavailability. Complementarily, the aqueous solubility of MP, expressed as LogS, was found to lie within the recommended range of 0 to − 6, which encompasses approximately 95% of approved drugs [102]. Therefore, MP may be considered moderately soluble, further supporting its potential pharmacokinetic adequacy.

The topological polar surface area (TPSA), a parameter derived from the contribution of polar atoms, specifically oxygen and nitrogen, and their attached hydrogens, provides an index of a molecule’s cellular permeability and transport capacity [103, 104]. A lower TPSA facilitates passive diffusion across lipid barriers, including the blood-brain barrier (BBB) and the gastrointestinal tract (GIT) [105]. Excessively high TPSA values, conversely, have been linked to reduced drug bioactivity of potential drugs due to impaired membrane penetration [106]. The TPSA value for MP was 85.22 Å², which is substantially below the threshold of < 140 Å² (Table 1), thereby indicating strong permeability characteristics consistent with drug-like behavior.

Hydrogen bonding capacity further influences solubility and permeability. Because hydrogen bonds must be disrupted to enable traversal through lipid bilayers, an excessive number may hinder passive diffusion [107]. Lipinski’s criteria recommend a maximum of 10 hydron bond acceptors (HBA) and 5 hydrogen bond donors (HBD) for optimal oral bioavailability [94]. The findings in Table 1 demonstrate that MP meets these criteria, underscoring its suitability fo effective absorption and systemic distribution.

**Fig. 2:**
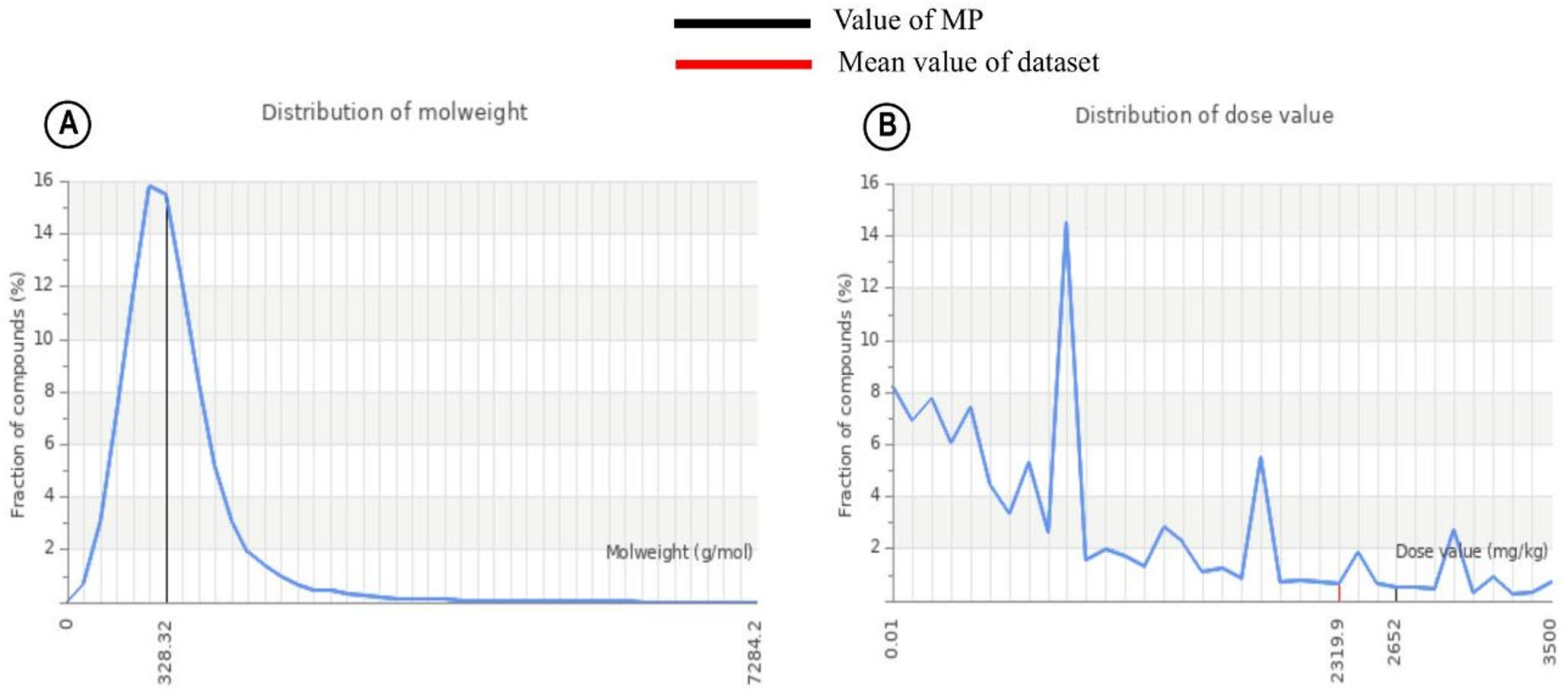
A graphical representation shows a comparison of MP with ProTox-II’s dataset compounds. **A** distribution of molecular weight, and **B** distribution of dose value

The medial lethal dose (LD_50_) refers to the median lethal dosage, indicating the quantity of a substance that results in the mortality of 50% of test subjects upon exposure and expressed in mg/kg body weight [108]. MP is classified as toxicity class 5 (2000 < LD_50_ ≤ 5000) and has an LD_50_ of 2652 mg/kg, according to the globally harmonized system of classification of labelling of chemicals [33, 34]. The toxicity assessment indicates that MP falls under toxicity class five (V), signifying less toxicity upon ingestion. Therefore, the MP is considered with comparatively low acute toxicity (Fig. 2).

### Target identification

SwissTargetPrediction predicted 100 potential molecular targets for MP, ranked according to a composite score that integrates both two-dimensional (2D) and three-dimensional (3D) similarity values relative to the most similar structurally known active compound. Complementarily, the SuperPred database predicted 115 prospective targets for MP, each assigned a probability score quantifying the likelihood of molecular binding based on advanced machine learning algorithms. Additionally, the PASS Online web resource identified 127 potential targets, which were evaluated through a confidence metric representing the probability differential between target engagement and non-engagement. Indeed, a higher confidence value reflects an increased probability of true positive interaction.

**Fig. 3:**
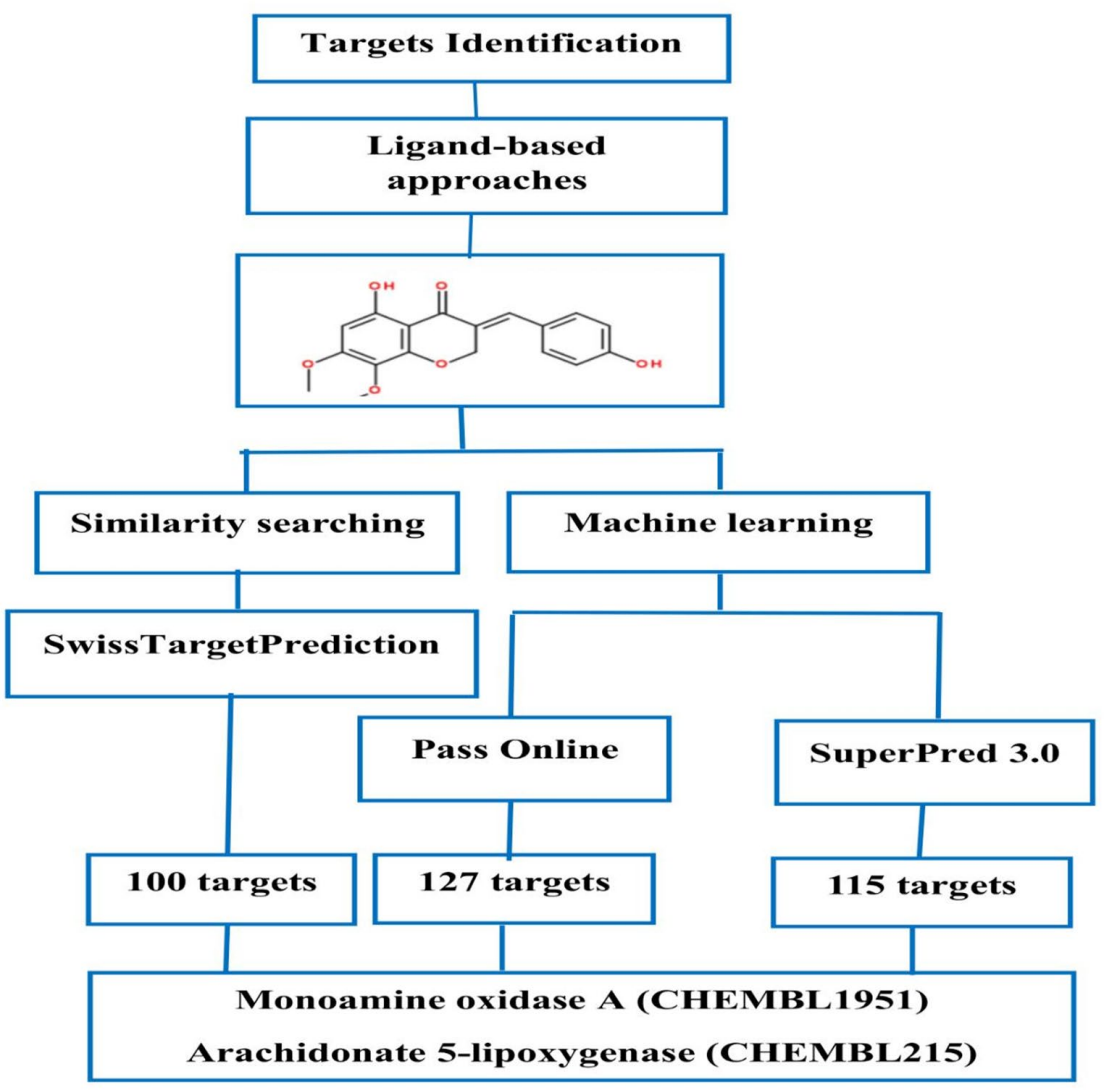
Flowchart of target prediction process using ligand-based approaches

**Fig. 4:**
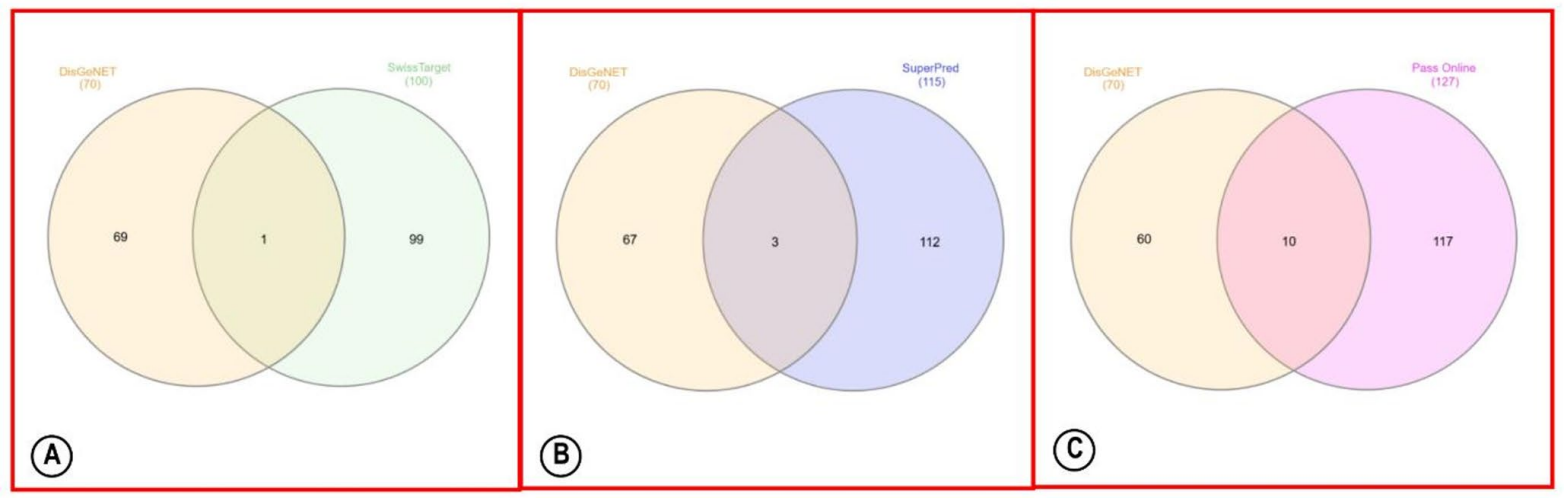
Venn diagram analysis for MP towards atherosclerosis disease genes using DisGeNET and **A** SwissTargetPrediction, **B** SuperPred and **D** Pass online

To ensure robustness, the datasets from all three platforms were consolidated, and only the overlapping targets common to all were retained. This stringent intersectional analysis revealed two consistent targets: Monoamine oxidase A (CHEMBL1951) and Arachidonate 5-lipoxygenase (CHEMBL215) (Fig. 3). Subsequently, seventy genes associated with atherosclerosis (Atherosclerosis, C0004153) were retrieved from the DisGeNET database. Comparative Venn analysis revealed that ALOX5 was the shared gene between MP-associated targets and atherosclerosis-related genes (Fig. 4). Thus, ALOX5 was selected as the primary molecular candidate for further mechanistic exploration.

### Analysis of PPI network

PPI analysis provides an indispensable framework for delineating the molecular interplay among predicted targets. In the constructed PPI network, each node represents a gene symbol, while the connecting edges denote experimentally supported or computationally inferred interactions. The resulting PPI network comprised 11 nodes and 22 edges, resulting in a mean degree of 4, thereby identifying highly connected “hub” proteins.

**Fig. 5:**
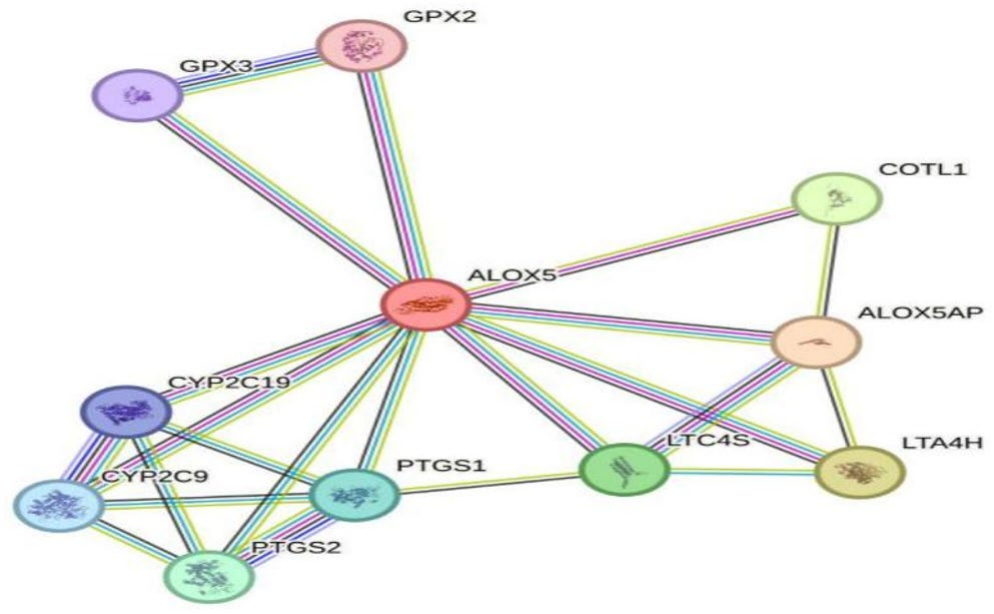
STRING PPI network for the top targets known by network analyser for MP as a SARS-ALOX5 inhibitor

Our findings indicated that the key biochemical signaling proteins involved in MP-associated PPIs include ALOX5, ALOX5AP, LTA4H, COTL1, LTC4S, PTGS2, PTGS1, CYP2C9, CYP2C19, GPX3, and GPX2 (Fig. 5). These nodes, by virtue of their elevated degree and betweenness values, are presumed to represent central mediators within the atherosclerotic signaling milieu, potentially constituting MP’s principal pharmacological interface.

### Identify the major hub proteins of the DEGs

**Fig. 6:**
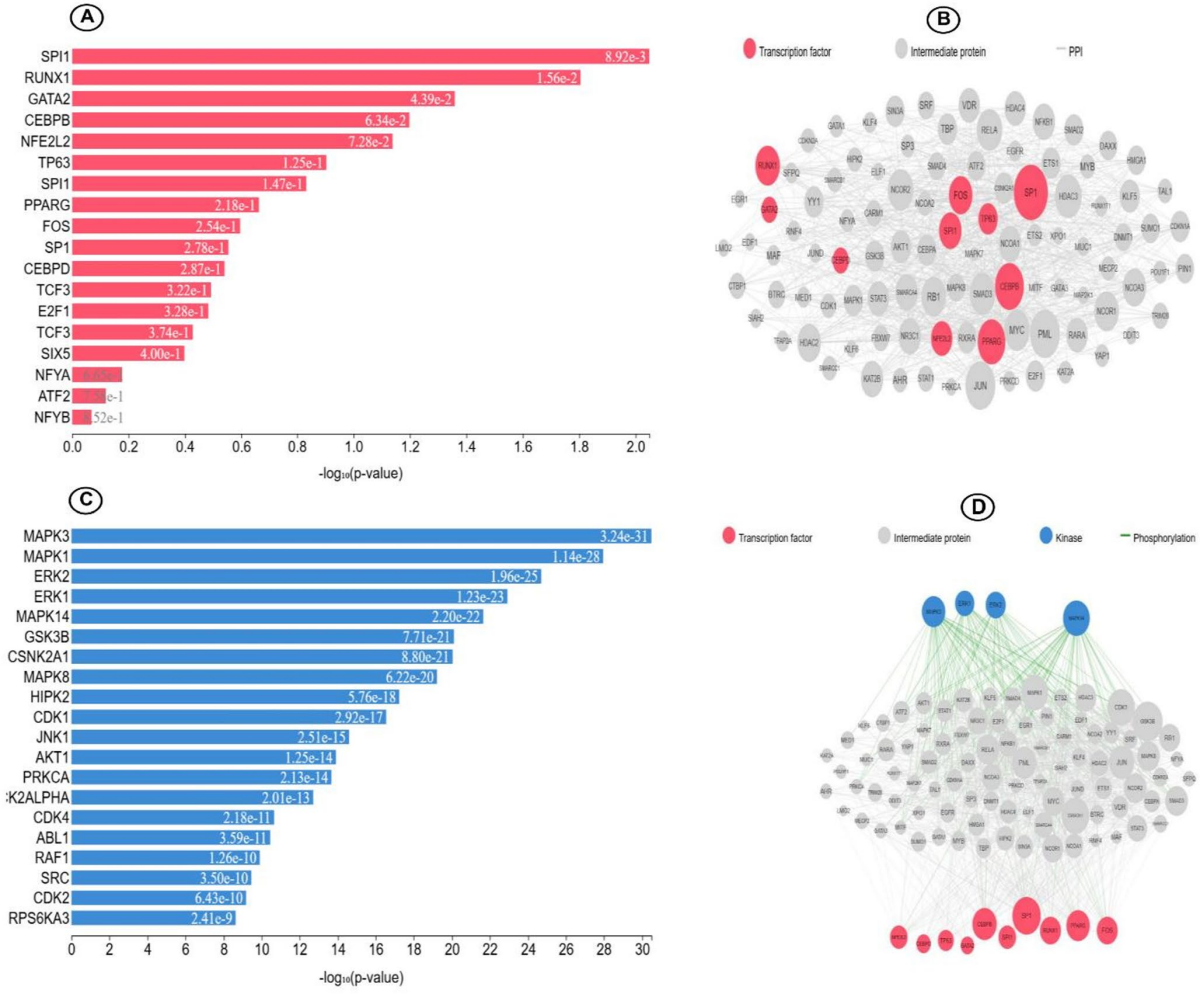
eXpression2Kinases tool for the analysis of **A** transcription factor enrichment analysis (TFEA), **B** protein–protein interaction expansion, **C** kinase enrichment analysis (KEA), and **D** eXpression2Kinases network

TFEA identified ten key transcription factors with significant predictive value among the DEGs: RUNX1, GATA2, CEBPB, NFE2L2, TP63, SP11, PPARG, FOS, SP1, and CEBPD ( Fig. 5A and B). Furthermore, integrated analysis combining TFEA and KEA revealed a close regulatory nexus between transcription factors and kinases, predominantly MAPK3, ERK1, ERK2, and MAPK14. These kinases exhibited extensive connectivty with the aforementioned transcription factors (i.e.RUNX1, GATA2, CEBPB, NFE2L2, TP63, SP11, PPARG, FOS, SP1, and CEBPD), delineating a coordinated signalling architecture (Fig. 6).

### Molecular docking analysis

**Fig. 7:**
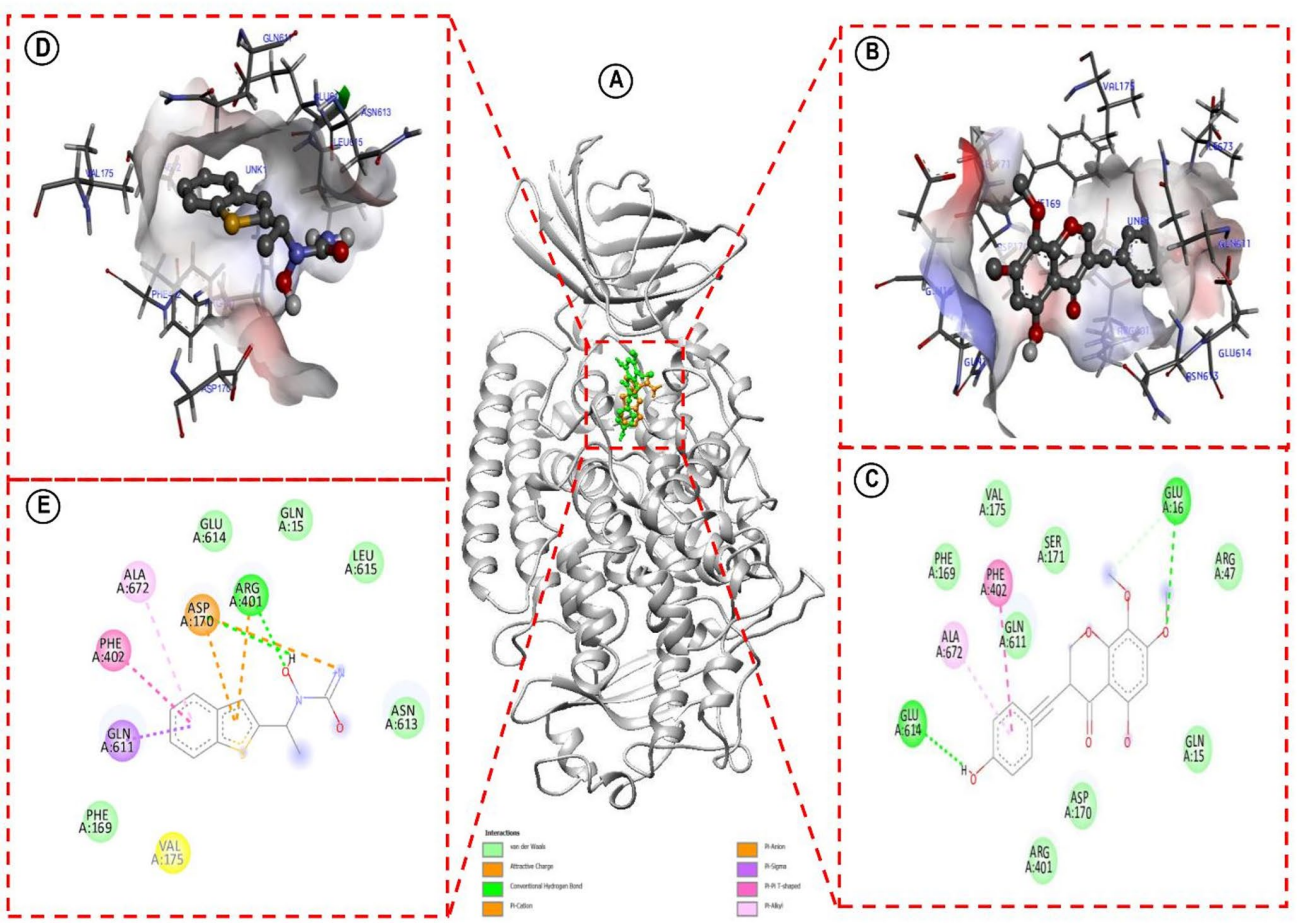
**A** predicted docking poses of MP (green) and Zileuton (orange) docked with ALOX5 enzyme. surface view of catalytic pocket of the ALOX5 enzyme and 3D visualization of the predicted docking pose of MP and Zileuton, **B** and **D** respectively. 2D visualization of the interaction landscape of docked MP and Zileuton complexes with ALOX5, **C** and **E** respectively

Molecular docking and subsequent MD simulations were conducted to investigate the binding interactions between MP and ALOX5. The docked complexes of ALOX5-MP and ALOX5-Zileuton exhibited substantial binding affinities, with docking scores of − 6.3 kcal/mol and − 6.5 kcal/mol, respectively. Both ligands formed multiple stabilizing interactions, including hydrogen bonding, π–π stacking, π-alkyl, π-anion, and π-sigma interactions. In the MP-ALOX5 complex, MP established hydrogen bonds with GLU16 and GLU614, π–π interactions with PHE402, and π-alkyl interactions with ALA672 (Fig. 7). Conversely, Zileuton formed hydrogen bonds with ASP170 and ARG401, along with π–π interactions (PHE402), π-alkyl (ALA672), π-anion (ASP170 and ARG401), and π-sigma (GLN611) interactions (Fig. 7). Consequently, it may be inferred that MP and Zileuton effectively engage the catalytic pocket of ALOX5 through analogous non-covalent forces, primarily mediated by the residues GLU16, ASP170, ARG401, PHE402, GLN611, GLU614, and LA672, conferring stable and specific enzyme-ligand complexes.

### Thermodynamics profile of MP and Zileuton

The MM/GBSA approach was employed to calculate the binding free energies of MP and Zileuton with ALOX5, offering atomic-level insight into their thermodynamic stability (Table 2). These computed energies provide evidence at the molecular level and could thus serve as the basis for developing small molecules that target ALOX5 with improved ligand binding characteristics. Importantly, MP exhibited a markedly more favorable total binding free energy (ΔG_bind_ = − 21.76 kcal/mol) than Zileuton (ΔG_bind_ = − 10.83 kcal/mol), underscoring its superior binding propensity.

The decomposition of energy components revealed that van der Waals forces (− 26.53 kcal/mol) and electrostatic interactions (− 19.31 kcal/mol) substantially contributed to MP binding, whereas solvation effects were mildly unfavorable. Indeed, these findings suggest that the energetics of MP-ALOX5 complexation are primarily driven by nonpolar interactions and intramolecular complementarity, providing a rational basis for its enhanced stability within the enzyme pocket.

**Table 2:** Free energy profile of MP and Zileuton binding to ALOX5

Systems	Energy components (kcal/mol)				
	ΔE_vdw_	ΔE_ele_	ΔG_gas_	ΔG_sol_	ΔG_bind_
ALOX5–MP complex	− 26.53 ± 0.12	− 19. 31 ± 0.19	− 45.85 ± 0.27	24.09 ± 0.16	− 21.76 ± 0.15
ALOX5–Zileuton complex	− 7.74 ± 0.18	− 6.20 ± 0.21	− 13.94 ± 0.35	8.11 ± 0.22	− 10.83 ± 0.15

All energies are in kcal/mol

ΔE_ele_ = electrostatic energy; ΔE_vdw_ = van der Waals energy; ΔG_bind_ = total binding free energy; ΔG_sol_ = solvation free energy; ΔG_gas_ = gas phase free energy

### Per-residue binding contribution analysis

**Fig. 8:**
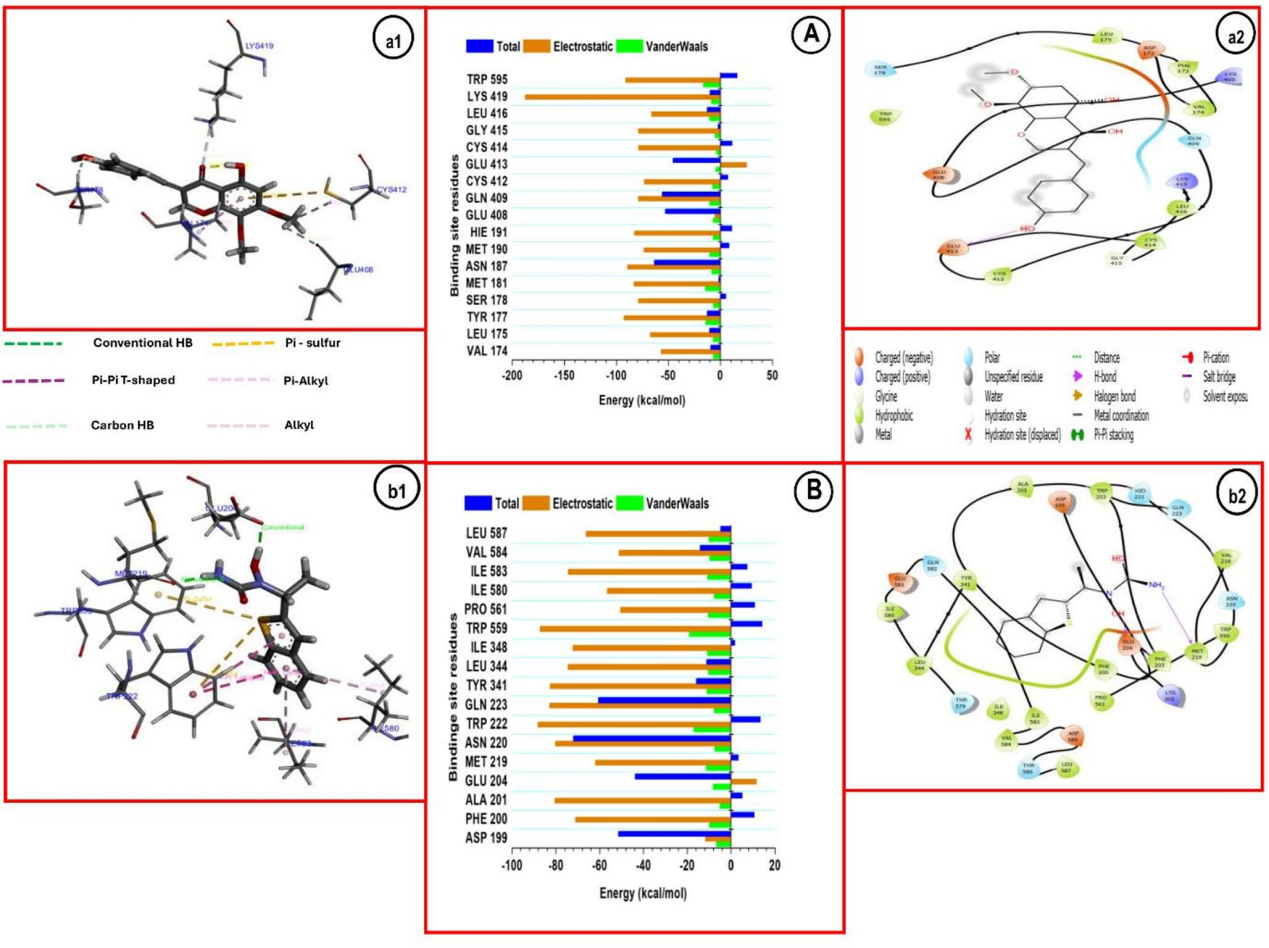
**A** and **B**: Graphical representation of Per-residue energy contribution of binding site residues towards MP and Zileuton complexing with ALOX5, respectively. **a**_**1**_ and **b**_**1**_: 3D model of the interactions between the MP and Zileuton at the binding site of ALOX5, respectively. **a**_**2**_ and **b**_**2**_: 2D model of the interactions between the MP and Zileuton at the binding site of ALOX5, respectively

The decomposition of the total free binding energies into individual contributions from each residue provides insightful understanding of the energetic architecture governing receptor-ligand interactions. Indeed, per-residue energy decomposition (PRED) analysis delineates which amino acid residues contribute most substantially to the stabilization of the complex, thus revealing the energetic raison d’etre of binding specificity. In this analysis, the total binding energies, comprising electrostatic, van der Waals, polar solvation, and nonpolar solvation components, were evaluated for each residue constituting the ALOX5 binding site. As shown in Fig. 8A, residues ASN187 (− 63.60 kcal/mol), GLN409 (− 55.99 kcal/mol) and GLU408 (− 52.92 kcal/mol) exhibited the most pronounced energetic contributions to MP-ALOX5 complex formation. Likewise, within the ALOX5-Zileuton complex, ASN220 (− 71.99 kcal/mol), GLN223 (− 60.65 kcal/mol), and ASP199 (− 51.42 kcal/mol) emerged as the principal contributors to binding stabilization (Fig. 8B). Owing to their dominant energetic participation, asparagine (ASN) and glutamine (GLN) residues appear to serve as pivotal mediators in driving the interactions of both MP and Zileuton within the ALOX5 active site. Their recurrent involvement in polar stabilization underscores their indispensable role in maintaining the binding pocket’s conformational integrity and intermolecular complementarity.

### Analysis of the structural landscapes associated with the binding of MP and Zileuton to ALOX5

By virtue of their intrinsic sensitivity to environmental perturbation, proteins often undergo conformational rearrangements upon ligand binding. The structural conformation of a protein is the very substrate of its functional properties; thus, alterations to its the structural integrity could significantly impact their biological activity. Understanding how small molecules such as MP and Zileuton reshape the conformational landscape of ALOX5 is therefore central to elucidating their inhibitory mechanisms.

**Fig. 9:**
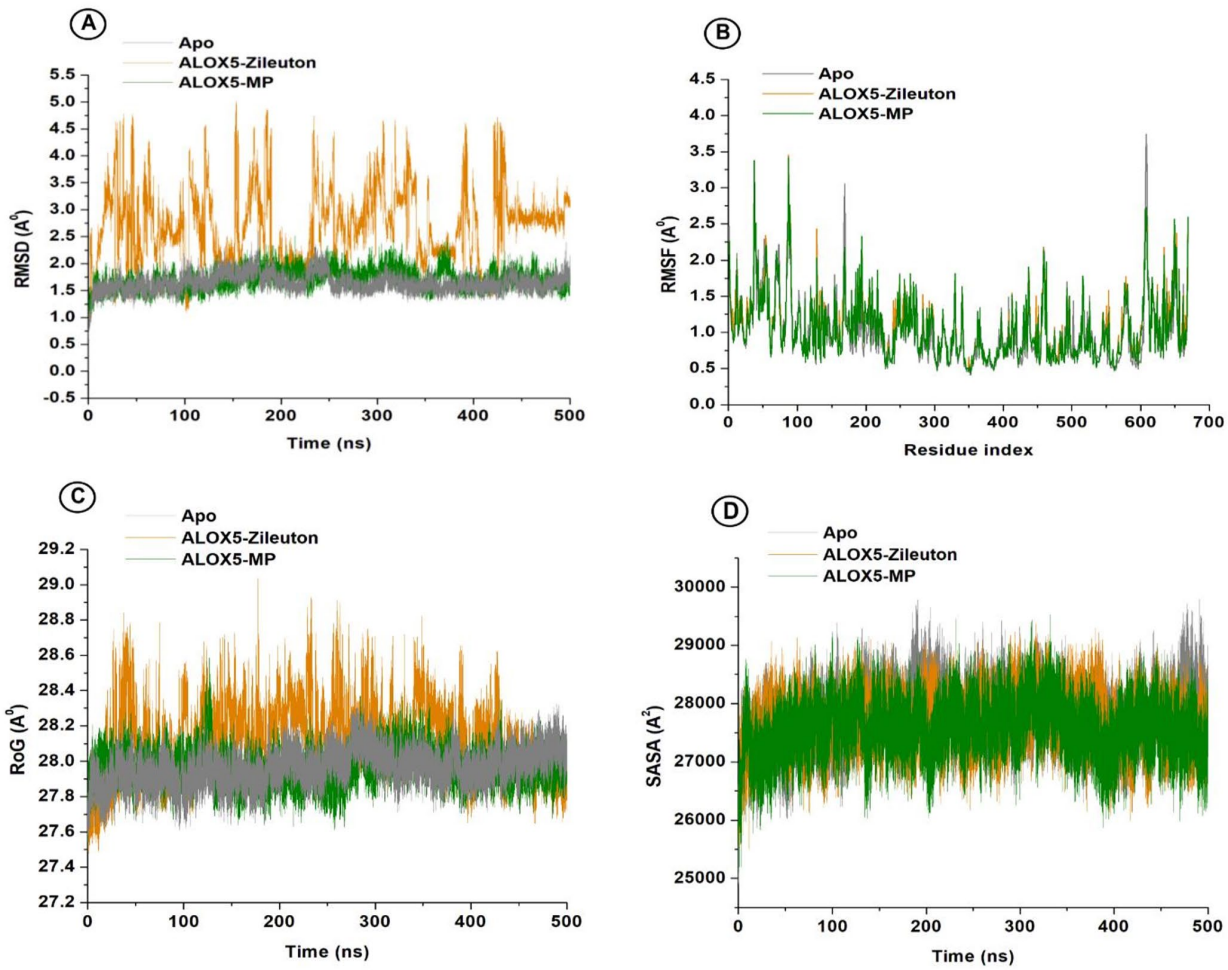
Graphical representation of RMSD **A**, RMSF **B**, RoG **C**, and SASA **D** values across Cα of ALOX5-Apo (grey), ALOX5-Zileuton complex (orange), and ALOX5-MP complex (green) over 500 ns MD simulations

To explore these structural dynamics, we first evaluated the Root Mean Square Deviation (RMSD) of the backbone atoms in unbound ALOX5 (apo form) and in the ALOX5-MP and ALOX5-Zileuton complexes. As shown in Fig. 9A, the average RMSD values were 1.70 Å for ALOX5-MP, 2.70 Å for ALOX5-Zileuton, and 1.64 Å for apo ALOX5. The increased deviations in the ligand-bound systems indicate that both MP and Zileuton induce measurable structural rearrangements within ALOX5, reflecting a shift toward alternative equilibrium conformations.

Further insight into local residue mobility was obtained through RMSF analysis, which quantifies atomic flexibility across residues. As shown in Fig. 9B, average RMSF values for ALOX5–MP, ALOX5–Zileuton, and apo forms were 1.05 Å, 1.08 Å, and 1.02 Å, respectively. These findings suggest that ligand binding subtly enhances the dynamic flexibility of ALOX5, potentially facilitating adaptive fit within the catalytic pocket. .

The conformational dynamics of ALOX5 upon binding to MP and Zileuton were further evaluated by calculating RoG to assess protein compactness. This metric was derived by computing the mass-weighted root mean square distance of selected atoms from the complex’s center of mass throughout MD simulations. The RoG values for the apo form, ALOX5-MP and ALOX5-Zileuton complexes were 27.96 Å, 27.97 Å and 28.13 Å, respectively (Fig. 9). The minimal differences between the apo and ALOX5-MP indicate only slight structural fluctuations, suggesting that MP binding induces minor compactness changes in ALOX5. These data imply a relatively stable overall folding upon ligand engagement.

To complement this, SASA analyses were used to characterize molecular mobility in terms of exposure to solvent environments. SASA quantifies the extent of hydrophilic and hydrophobic surface interactions, where elevated SASA values reflect increased protein surface exposure to solvent and lower values indicate a more occluded, hydrophobic environment. Throughout the simulation, the Apo system consistently exhibited higher SASA readings, with an average of 27788.19 Å^2^ (Fig. 9). Conversely, the complexes with MP and Zileuton maintained lower SASA values—averaging 27,557.90 Å² and 27,673.92 Å², respectively—indicating that ligand binding promotes a more compact, folded conformation of ALOX5. Collectively, these observations suggest that MP and Zileuton’s interactions alter ALOX5’s global dynamics, reinforcing its structural stability.

### Hydrogen bond analysis

Intramolecular hydrogen bonding analysis provided further insight into the enzyme’s conformational stability and the nature of ligand interactions (Fig. 9). Evaluating hydrogen bonds yields critical information on the structural integrity of the ALOX5-ligand complexes and the stabilization mechanisms underpinning binding [109]. The average number of intramolecular hydrogen bonds observed was 350 for the ALOX5-MP complex and 353 for the ALOX5-Zileuton complex (Fig. 9A). Three-dimensional representations of hydrogen bonding within the active site highlighted distinct donor and acceptor interactions for MP and Zileuton (Fig. 9B and C), underscoring the molecular basis for ligand-specific conformational effects. These findings emphasize the role of hydrogen bonding networks in maintaining enzyme-ligand stability and functional conformation.

**Fig. 10:**
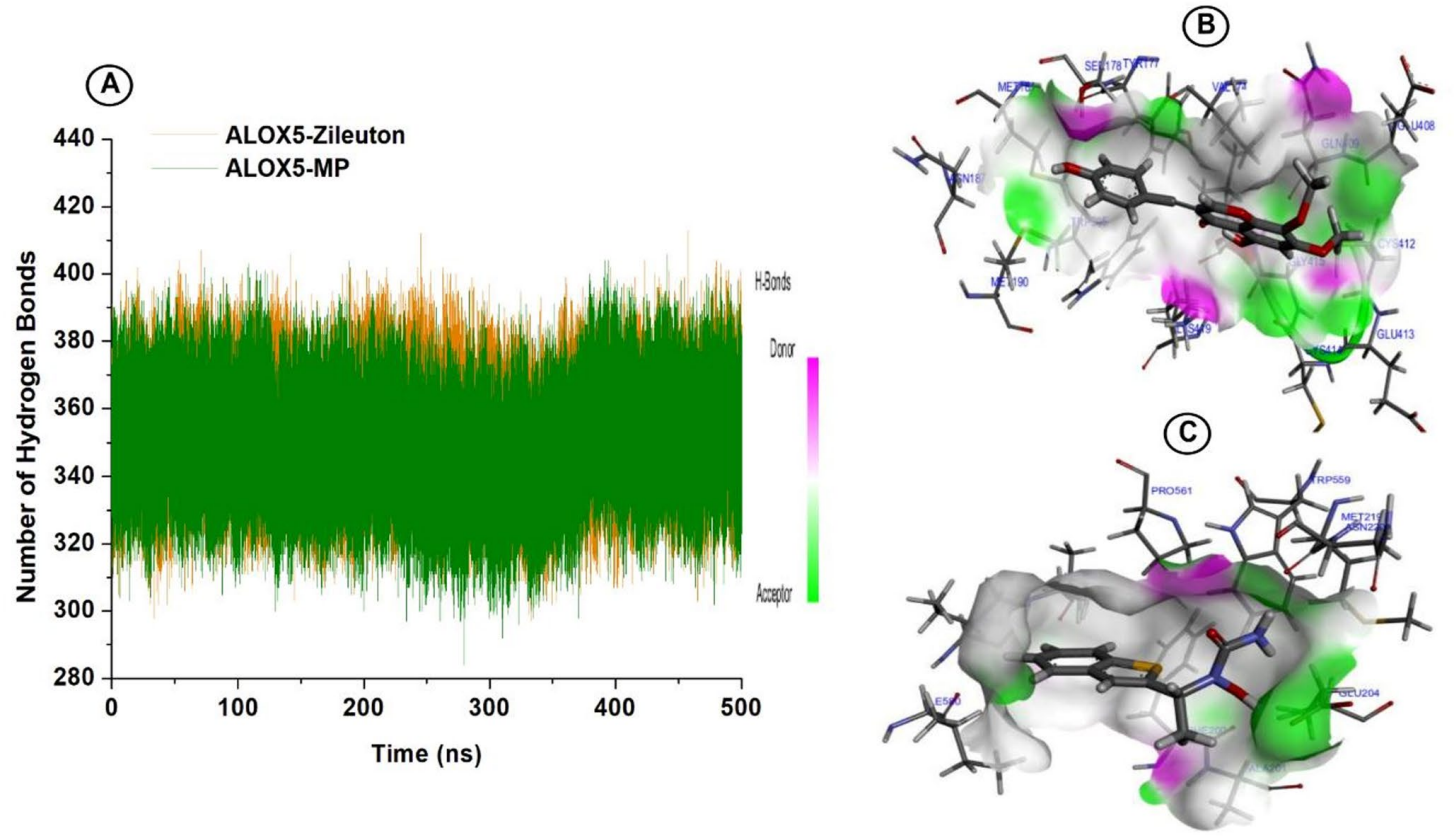
Hydrogen bond analysis. **A** Intramolecular hydrogen bonds in ALOX5 enzyme with the Zileuton (orang) and MP (green). 3D hydrogen bond representation within the active site of ALOX5 when bound to MP (**B**) and Zileuton (**C**). The degree of hydrogen bond donors (magenta) and acceptors (light green)

### DCCM plots

DCCM analyses were conducted to explore residue-level correlated and anti-correlated motions across the apo and ligand-bound states (Fig. 10). The most pronounced anti-correlated motions, indicated by the most negative regions (blue - black) matrix regions, contrast with the most positively correlated motions (orange–green) (Fig. 10). Both Apo and ALOX5-MP exhibited a mixture of positive and negative motion correlations throughout the simulation, reflecting a balanced dynamic landscape. Conversely, the ALOX5-Zileuton displayed a distinctive pattern characterized by stronger positive correlations, suggesting ligand-induced shifts toward coordinated residue motions (Fig. 10). This differential dynamic behavior provides mechanistic insight into how ligand binding modulates the internal communication and flexibility of ALOX5.

**Fig. 11:**
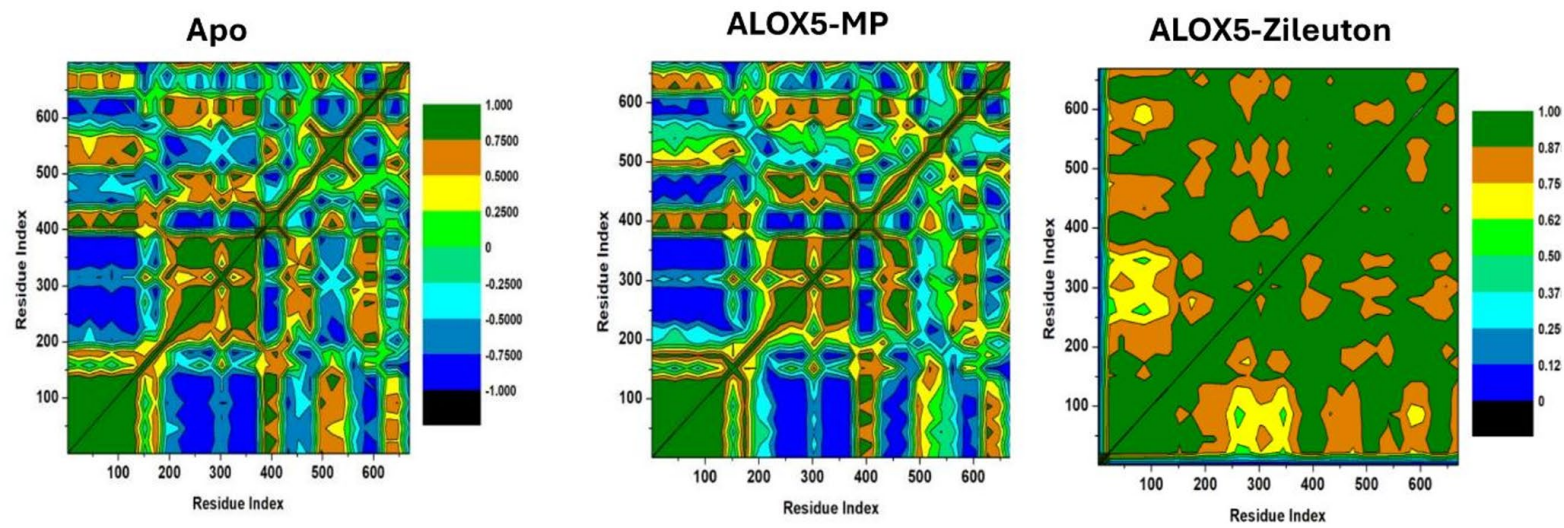
Shows dynamic cross-correlation matrix analyses for ALOX5 ligand-free state (Apo) and when bound to MP, and Zileuton calculated from the MD trajectories of 500 ns

### Investigation of motional dynamics via PCA

**Fig. 12:**
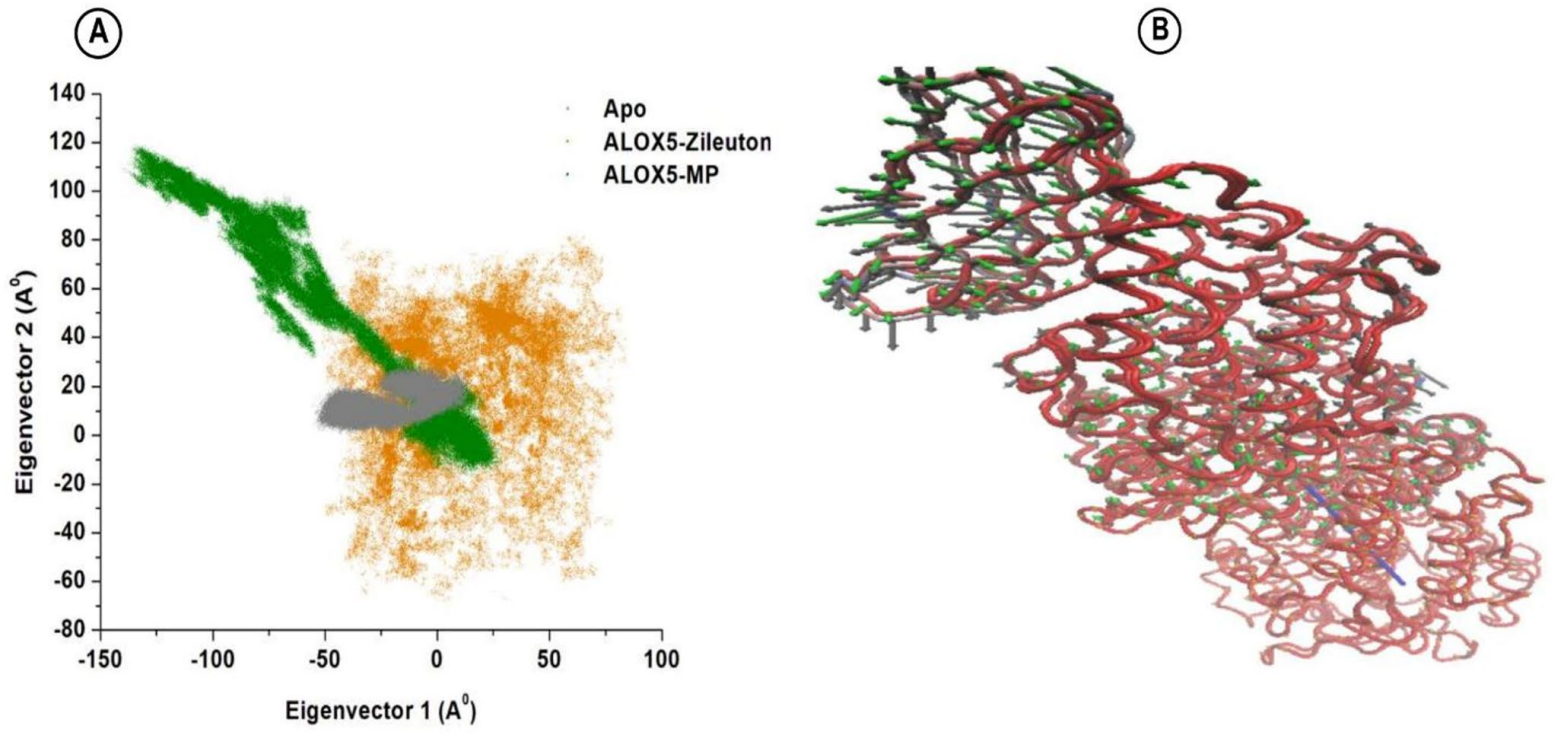
**A** Graphical representation of PCA for Apo (grey), ALOX5-Zileuton complex (orange), and ALOX5-MP complex (green). **B** Collective motions of PC1 for the obtained dominating EVs using PCA during the 500 ns MD simulations

The impact of ligand-induced binding on the main conformational changes of each residue was qualitatively investigated by comparing the intense movements of ALOX5 in unbound and when bound to MP and Zileuton with PCA utilizing the first two eigenvectors. The 2D scatter plots derived from PCA (Fig. 11A) reveal marked alterations in the overall motions when comparing the Apo enzyme to its ligand-bound counterparts. The ALOX5-Zileuton system depicts tightly correlated motions with reduced fluctuations, indicating a stabilized conformational state. In contrast, the ALOX5-MP complex displays pronounced anti-correlated fluctuations, reflected by expanded scatter patterns with negative values, highlighting increased dynamic heterogeneity. The Apo form retains a blend of correlated and anti-correlated motions, signaling intrinsic flexibility in the absence of ligand (Fig. 11A). Furthermore, transition state analyses (Fig. 11B) illustrate that the ALOX5-MP complex undergoes more frequent conformational excursions, implying an enhanced transition rate between structural states relative to both Apo and ALOX5-Zileuton systems. These dynamic differences underscore the ligand-specific modulation of ALOX5’s conformational plasticity and functional dynamics. (Fig. 12)

To strengthen the comparison between MP and the reference drug Zileuton, additional/replicate simulations were conducted and results were statistically analyzed. MP demonstrated a significantly stronger MM/GBSA binding free energy compared to Zileuton’s (–21.76 ± 0.15 kcal/mol versus − 10.83 ± 0.15 kcal/mol, respectively; *p* < 0.01). Dynamic analyses further supported this observation: MP exhibited a lower RMSD (1.70 Å versus 2.70 Å; *p* < 0.05) and a marginally more compact RoG (27.97 Å versus 28.13 Å; *p* < 0.05). Although RMSF values (1.05 Å versus 1.08 Å) and hydrogen bond counts (350 versus 353) were not statistically different, MP displayed a marginally reduced SASA (27,557.90 Å² versus 27,673.92 Å²; *p* < 0.05) (Table 3). Collectively, these findings indicate that MP forms a more stable, compact, and energetically favorable complex than Zileuton, reinforcing its potential as a promising inhibitor.

**Table 3:** Comparative MM/GBSA energies and dynamic properties of MP and Zileuton

Parameter	MP (mean ± SD)	Zileuton (mean ± SD)	*p*-value	Interpretation
MM/GBSA (kcal/mol)	− 21.76 ± 0.15	− 10.83 ± 0.15	< 0.01	MP shows significantly stronger binding affinity
RMSD (Å)	1.70 ± 0.10	2.70 ± 0.12	< 0.05	MP complex is more structurally stable
RMSF (Å)	1.05 ± 0.08	1.08 ± 0.09	n.s.	Flexibility is comparable
Radius of gyration (Å)	27.97 ± 0.18	28.13 ± 0.20	< 0.05	MP complex is slightly more compact
SASA (Å²)	27557.90 ± 135.6	27673.92 ± 142.3	< 0.05	MP complex has marginally reduced solvent exposure
Hydrogen bonds	35 ± 5	353 ± 6	n.s.	H-bonding patterns are similar

## Conclusion and future perspective

The identification of ALOX5 as a critical molecular target of MP advances the mechanistic understanding of its anti-atherosclerotic properties with precision and depth. MP’s modulation of ALOX5’s structural dynamics and interaction networks underscores its capacity to disrupt key pathogenic pathways in vascular pathology. These findings exemplify the power of integrating in silico molecular modeling with pharmacological profiling to reveal novel therapeutic avenues. Furthermore, the study positions MP as a promising agent capable of refining VSMC phenotypes and attenuating inflammation-driven atherogenesis through targeted enzyme inhibition.

The present findings highlight that ALOX5 functions within an intricate vascular signaling network shaped by oxidative, endothelial, and hormonal modulators. Endothelial nitric oxide bioavailability, mitochondrial efficiency, and estrogenic tone act in concert to define vascular responsiveness. The PI3K/Akt/eNOS/NO/cGMP pathway can serve, by virtue of its antioxidant and vasodilatory properties, as a natural counterbalance to ALOX5-driven oxidative signalling [110]. Moreover, mitochondrial preservation further supports this balance by maintaining redox homeostasis and preventing excessive ROS accumulation. Notably, adrenoceptor expression in VSMCs is upregulated by various endogenous ligands (e.g. estrogen) through redox-sensitive cAMP signaling—a mechanism that heightens vasoconstrictive tone under oxidative stress, especially given the role of estrogen in hypertension [111–119]. Because ALOX5 is a substantial enzymatic source of ROS, it may indirectly potentiate this estrogen–adrenoceptor interplay, linking inflammatory lipid signaling with adrenergic hyperreactivity. Owing to this convergence of oxidative, hormonal, and endothelial mechanisms, ALOX5 inhibition by 7MP may restore vascular equilibrium across multiple regulatory layers, representing not merely an anti-inflammatory intervention but the very *raison d’être* of its vasoprotective profile.

The implications of MP’s selective engagement with ALOX5 reach far beyond foundational biochemistry. They compel a re-examination of how natural compounds might be leveraged to modulate complex enzyme functions within inflammatory cascades driving atherosclerosis. Yet, critical steps remain. Indeed, translating these in silico findings into physiologically relevant outcomes requires robust animal model validation, encompassing pharmacokinetic behavior, toxicity, and therapeutic efficacy under clinically relevant conditions. Concurrently, a better understanding of MP’s effect on interconnected signaling pathways will likely highlight its broader vascular effects, potentially uncovering synergistic targets to amplify cardiovascular protection. Structural elucidation through techniques like cryo-EM will be vital in refining MP derivatives with superior binding and bioavailability. Ultimately, advancing MP from computational promise to clinical reality demands interdisciplinary efforts integrating molecular pharmacology, medicinal chemistry, and translational medicine, aiming to curb the global burden of cardiovascular disease through innovative, targeted therapies.

## Data Availability

Data is available upon reasonable request.

## Abbreviations

ALOX5: Arachidonate 5-lipoxygenase
CVD: cardiovascular disease
DEGs: differentially expressed genes
HB: Hydrogen bonds
KEA: Kinase enrichment analysis
LDL: low-density lipoprotein
LD_50_: lethal dose
MP: 7-O-methylpunctatin
PPI: protein–protein interactions
PRED: per-residue energy decomposition
RMSF: Root mean square fluctuation
RMSD: Root mean square deviation
RoG: Radius of gyration
SASA: solvent-accessible surface area
TFEA: Transcription factor enrichment analysis
TPSA: The topological polar surface area
VSMC: vascular smooth muscle cell
